# Repression of Polyol Pathway Activity by *Hemidesmus indicus* var*. pubescens R.Br. Linn* Root Extract, an Aldose Reductase Inhibitor: An In Silico and Ex Vivo Study

**DOI:** 10.1007/s13659-020-00290-w

**Published:** 2020-12-07

**Authors:** Hajira Banu Haroon, Vijaybhanu Perumalsamy, Gouri Nair, Dhanusha Koppal Anand, Rajitha Kolli, Joel Monichen, Kanchan Prabha

**Affiliations:** 1 Department of Pharmacology, Faculty of Pharmacy, M S Ramaiah University of Applied Sciences, Gnanagangothri Campus, New BEL Road, Bengaluru, Karnataka 560054 India; 2 Department of Pharmaceutical Chemistry, Faculty of Pharmacy, M S Ramaiah University of Applied Sciences, Gnanagangothri Campus, New BEL Road, Bengaluru, Karnataka 560054 India; 3 Department of Pharmaceutics, Faculty of Pharmacy, M S Ramaiah University of Applied Sciences, Gnanagangothri Campus, New BEL Road, Bengaluru, Karnataka 560054 India

**Keywords:** *Hemidesmus indicus* var. *pubescens*, Aldose reductase, Polyol pathway, Diabetic cataract

## Abstract

**Abstract:**

Development of diabetic cataract is mainly associated with the accumulation of sorbitol via the polyol pathway through the action of Aldose reductase (AR). Hence, AR inhibitors are considered as potential agents in the management of diabetic cataract. This study explored the AR inhibition potential of *Hemidesmus indicus* var. *pubescens* root extract by in silico and ex vivo methods. Molecular docking studies (Auto Dock tool) between β-sitosterol, hemidesminine, hemidesmin-1, hemidesmin-2, and AR showed that β-sitosterol (− 10.2 kcal/mol) and hemidesmin-2 (− 8.07 kcal/mol) had the strongest affinity to AR enzyme. Ex vivo studies were performed by incubating isolated goat lenses in artificial aqueous humor using galactose (55 mM) as cataract inducing agent at room temperature (pH 7.8) for 72 h. After treatment with Vitamin E acetate − 100 µg/mL (standard) and test extract (500 and 1000 µg/mL) separately, the estimation of biochemical markers showed inhibition of lens AR activity and decreased sorbitol levels. Additionally, extract also normalized the levels of antioxidant markers like SOD, CAT, GSH. Our results showed evidence that *H. indicus* var. *pubescens* root was able to prevent cataract by prevention of opacification and formation of polyols that underlines its potential as a possible therapeutic agent against diabetic complications.

**Graphic Abstract:**

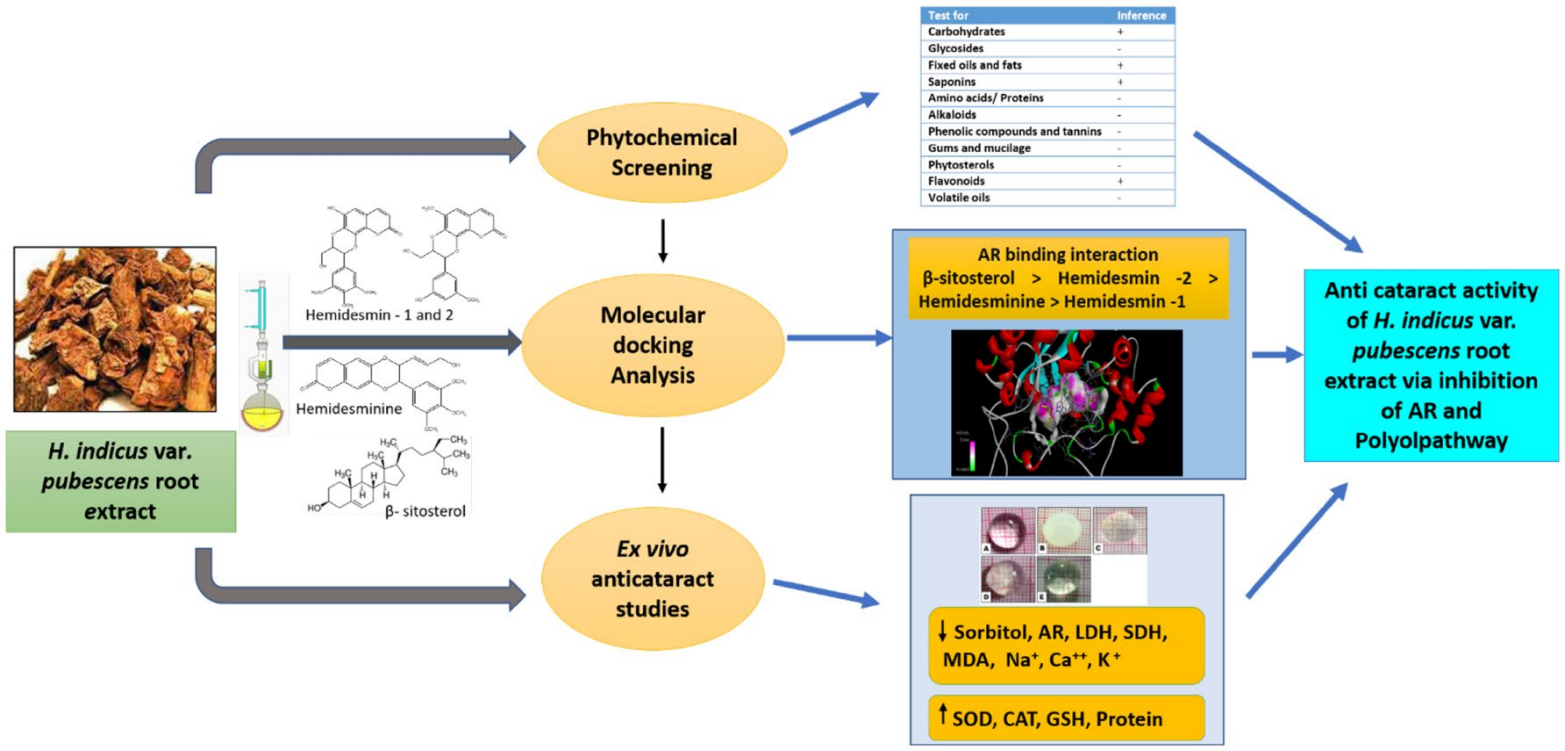

## Introduction

Cataract has become one of the leading causes of visual impairment and blindness worldwide and diabetes mellitus is considered to be one of the major risk factors. As the prevalence of diabetes is increasing, the incidence of diabetic cataract has also risen and about 20–30% of cataract surgeries are performed on diabetic patients alone. A cataract is the opacification or optical dysfunction of the crystalline lens, associated with the breakdown of the eye lens micro-architecture, which interferes with the transmission of light onto the retina [1]. In Diabetes Mellitus, the cellular levels of glucose greatly increase in tissues where glucose entry is independent of insulin, like the lens, retina, kidney, and peripheral nerves. Due to this extra pressure on the lens, it becomes inflexible and this damages cells to the point of cataract formation. Hyperglycemia or sustained increase of blood glucose contributes to cataract formation in three ways viz., non-enzymatic glycation of eye lens proteins, activated polyol pathway in glucose disposition, and oxidative stress [2].

Aldose reductase, an important enzyme in the polyol pathway catalyzes the reduction of glucose to sorbitol, intracellular accumulation of which leads to osmotic stress resulting in degeneration of lens fibers and cataract formation. This accumulation of polyol is also associated with the liquefaction of the lens resulting in the formation of lens opacities [3]. Therefore, AR activation is considered one of the most significant mechanisms that are responsible for the complication associated with diabetes mainly cataract. Many studies indicate that inhibition of AR could be effective in the treatment of diabetic cataract. Though several synthetic AR inhibitors (ARIs) are being introduced for managing diabetic complications, they are associated with low permeability to target tissues as well as have many side effects. Hence, studies are now being directed to identify potential ARIs from natural sources that could be beneficial in the treatment of diabetic complications with the least or no side effects. In this context, the present study was designed to determine the anti- cataract potential of *Hemidesmus indicus* var. *pubescens* via the inhibition of AR.

*Hemidesmus indicus* var. *indicus* is known as Indian Sarsaparilla [4]. In Ayurveda, it is known as *Sariva* and is used to treat venereal and skin diseases, arthritis, rheumatism, epilepsy, nervous disorders, tonsillitis, liver disease and syphilis, stomach disorders and as an aphrodisiac to treat impotence [5, 6]. These activities are accounted to the presence of a number of phytochemicals such as 2-hydroxy-4-methoxy benzaldehyde, salicylaldehyde, limonene, methyl salicylates, isovanillin, β-amyrin acetate, terpenoids, β-sitosterols, hemidesmine (coumarinolignoid), hemidesmin-1 and 2, β-amyrin palmitate, hemidesmusoic acid, hemidesmus ester, terpenoic ester, salicylic acid derivative, etc. [7].

Many pharmacological studies have also been reported on this plant-like *H. indicus* var. *indicus* is reported to have anti-diabetic property. *Hemidesmus indicus* (L.). R.Br. var. *pubescens* (Wight & Arn) Hk.f. is a taxonomic variety found in south India proven to have antioxidant property [8, 9], anti-microbial [10], antipyretic and antiepileptic activity [11], and hepatoprotective activity [12]. Since no study has been reported on the anticataract potential of *Hemidesmus indicus* var. *pubescens,* the present study was undertaken.

## Results

### Phytochemical Analysis

Preliminary phytochemical analysis of the alcohol extract was performed to detect the presence of primary and secondary metabolites. The extract revealed the presence of carbohydrates, fixed oils, and fats, saponins, and flavonoids (Table 1).

**Table 1:** Preliminary phytochemical screening of *H. indicus* var. *pubescens* root extract

Sl no	Test for	Inference
1	Carbohydrates	+
2	Glycosides	−
3	Fixed oils and fats	+
4	Saponins	+
5	Amino acids/proteins	−
6	Alkaloids	−
7	Phenolic compounds and tannins	−
8	Gums and mucilage	−
9	Phytosterols	−
10	Flavonoids	+
11	Volatile oils	−

+ Present; − absent

### Molecular Docking Studies

AutoDock 1.5.6 study results (Table 2) showed that β-sitosterol, hemidesminine, hemidesmin—1, and 2 interacted with amino acid residues of the protein. β-sitosterol exhibited the highest binding energy (− 10.2 kcal/mol) on AR by interacting with Lys 262, Tyr 209, Trp 20, Trp 111, Trp 219, Cys 298, Ile 260, His 110, Leu 300, Phe 122 with characteristic pi-alkyl, pi sigma and alkyl interactions. Hemidesmin-2 showed the second-highest binding energy (− 8.07 kcal/mol) with AR making hydrogen bonds, pi-pi stacking, alkyl, pi-alkyl interactions with amino acid residues like Tyr 48, Val 47, Trp 20, Asp 43, Lys 77, Asn 160, Ser 159, Cys 298, His 110. On the other hand, hemidesminine and hemidesmin—1 showed the lowest binding energy (− 5.86 and − 4.18 kcal/mol respectively) and were associated with conventional hydrogen bonds, Pi–Pi stacking, alkyl, pi-alkyl and pi–sigma interaction. Hemidesminine interacted with Pro 261, Pro 211, Trp 111, Cys 298, Ala 299, Leu 300, Trp 216, Pro 218, Trp 20, His 110, Tyr 209, Ile 260, Ser 210, Lys 262 of the AR protein while hemidesmin—1 interacted with Phe 122, Tyr 48, Lys 21, Trp 20, Ser 210, Tyr 209, Asp 43, Lys 77, Gln 183, Ser 159, Cys 298, His 110, Leu 300, Ala 299, Trp 219 of the AR protein. Therefore, the binding energy of major constituents of *H. indicus* with AR is in the order: β-sitosterol ˃ Hemidesmin-2 ˃ Hemidesminine ˃ Hemidesmin-1.

**Table 2:** Molecular docking studies

Receptor and PubMed ID	Ligand	Binding energy (− kcal/mol)	Interacting residues	Ligand efficiency
Aldose reductase (1US0)	β-sitosterol	− 10.2	LYS262, TYR209, TRP20, TRP111, TRP219, CYS298, ILE260 HIS110, LEU300, PHE122	− 0.34
	Hemidesminine	− 5.86	PRO261, PRO211, TRP111, CYS298, ALA299, LEU300, TRP216, PRO218, TRP20, HIS110, TYR209, ILE260, SER210, LYS262	− 0.19
	Hemidesmin -1	− 4.18	PHE122, TYR48, LYS21, TRP20, SER210, TYR209, ASP43, LYS77, GLN183, SER159, CYS298, HIS110, LEU300, ALA299, TRP219	− 0.14
	Hemidesmin-2	− 8.07	TYR48, VAL47, TRP20, ASP43, LYS77, ASN160, SER159,CYS298, HIS110	− 0.3

**Fig. 1:**
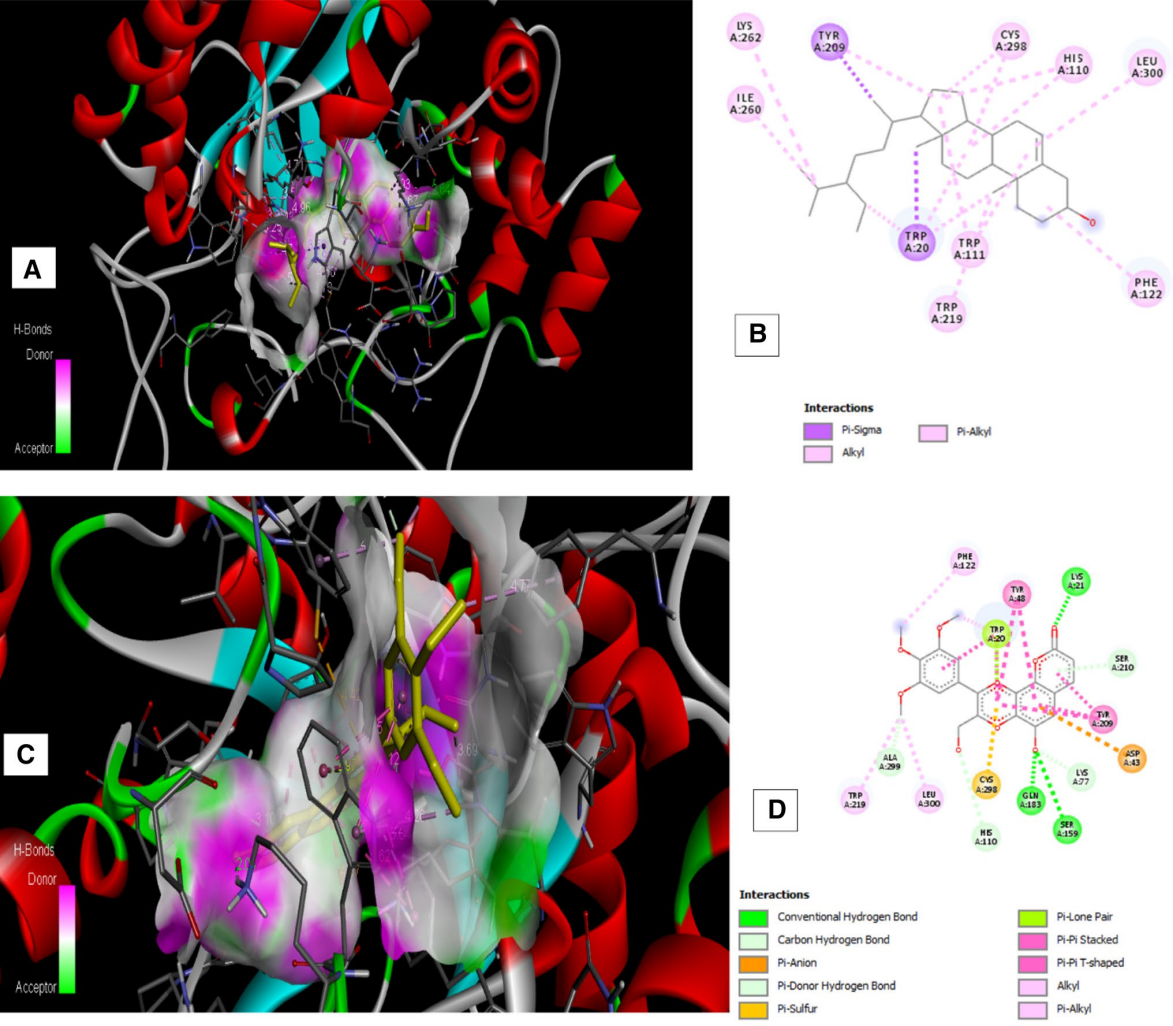

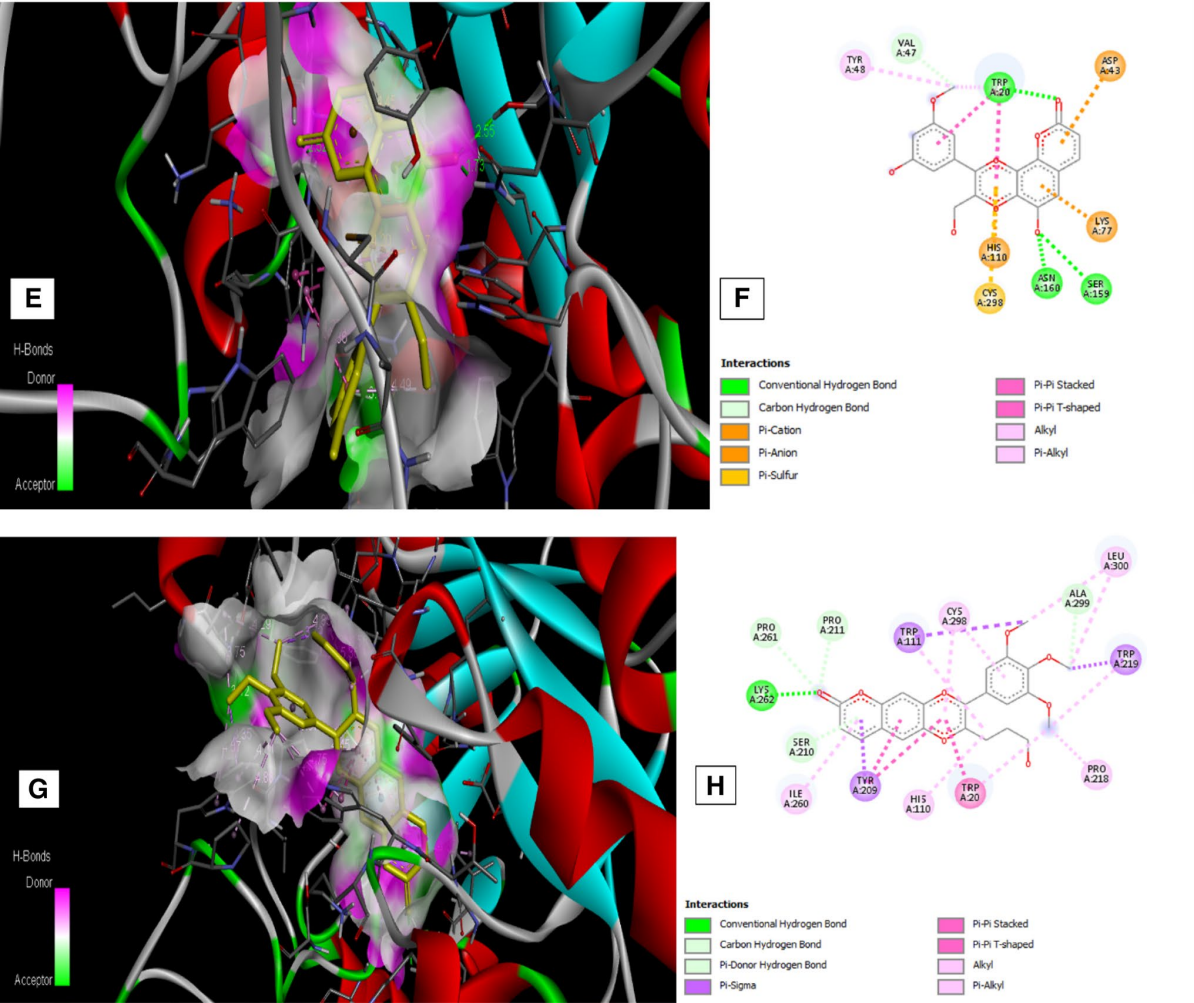
Molecular Interaction of compounds with AR protein. **a**, **b** Interaction of β-sitosterol with AR protein and its associated amino acid residues. **c**, **d** Interaction of Hemidesmin—1 with AR protein and its associated amino acid residues. **e**, **f** Interaction of Hemidesmin—2 with AR protein and its associated amino acid residues. **g**, **h** Interaction of Hemidesminine with AR protein and its associated amino acid residues

Ligand efficiency indicates the optimal ligand binding to protein that depends on the binding energy of the ligand. It is a sum measure of molecular properties like size, the lipophilicity of the molecule that are required to achieve binding affinity to a drug target. Ligand efficiency of 0.3 is considered sufficient for a compound to be a drug like to have a higher affinity with the target [13]. In this study, β-sitosterol, hemidesminine, hemidesmin—1, and 2 showed the ligand efficiency of − 0.34, − 0.19, − 0.14, and − 0.3 respectively. β-sitosterol and hemidesminine has higher ligand efficiency than hemidesmin—1 and 2 indicating optimal ligand binding capacity of β-sitosterol and hemidesminine with AR protein (Fig. 1).

### Lens Morphology

**Fig. 2:**
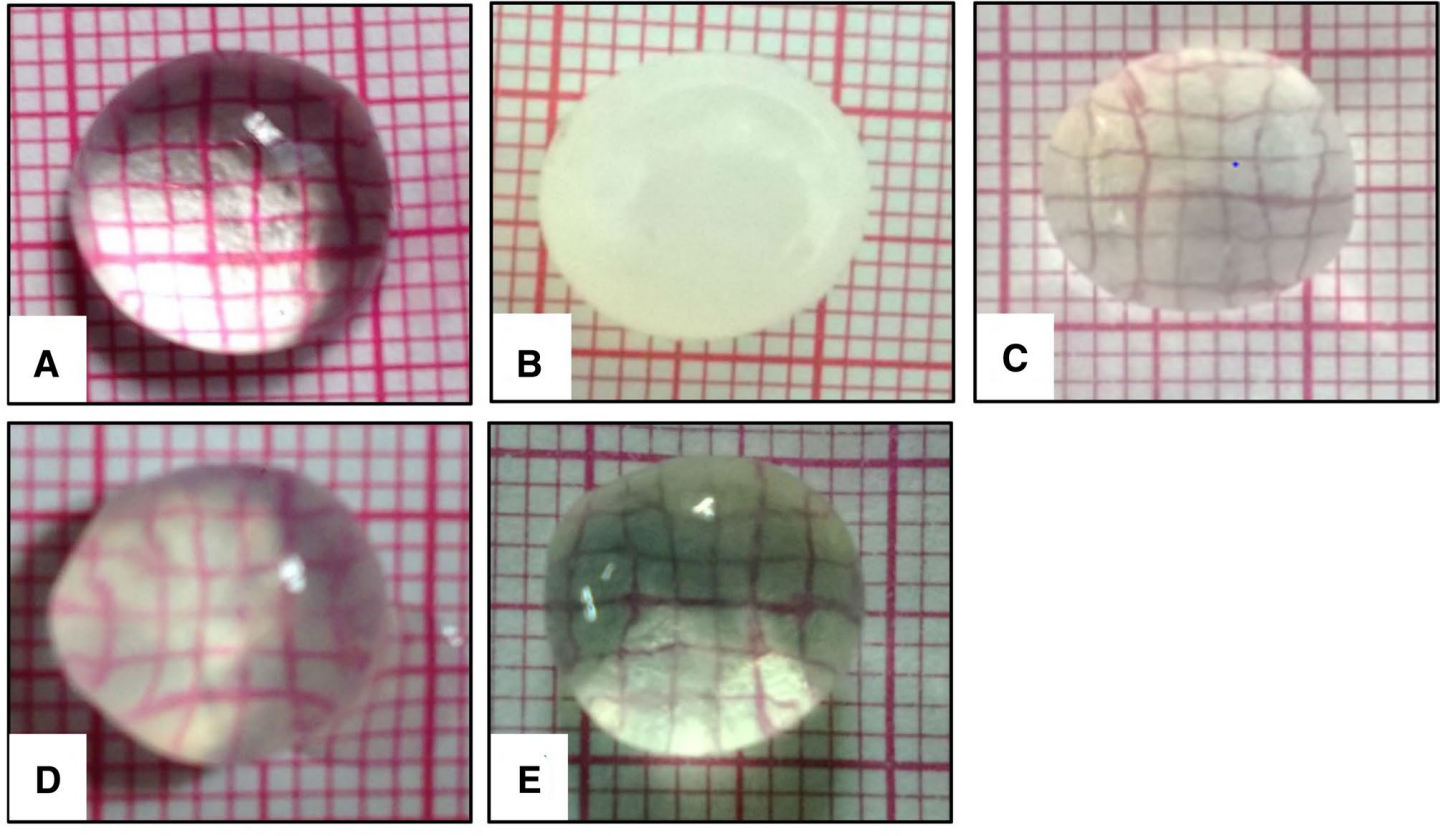
Effect of *H. indicus* on lens morphology. **a** Lens of normal control (Grade: 0 changes); **b** Lens of Positive control (Mature cataract) (Grade 4: changes); **c** Lens treated with standard Vitamin E acetate (100 µg/mL) (Grade-1 changes); **d** Lens treated with low Concentration of *H. indicus* extract (500 µg/mL) (Grade-2 changes); **e** Lens treated with high concentration of *H. indicus* extract (1000 µg/mL) (Grade-1 changes)

The lenses of normal control were transparent and clear through which grid lines of graph paper were visible. As compared to the lenses of normal control, the lenses in positive control showed complete loss of transparency (opaque lens) confirmed by the invisibility of grid lines and a swollen, matured cataract nearing rupture thus displaying Grade 4 changes. Treatment with standard and high concentration of extract had decreased the cataract development that was evidenced by the visible grid lines, minimal lens swelling, and intact lens shape hence both showing Grade 1 changes. However, the lenses treated with a low concentration of test extract showed faintly visible grid lines and slight swelling thus showing Grade 2 changes (Fig. 2).

### Lens Homogenate Analysis

The lens homogenate was subjected to biochemical analysis to determine the effect of *H. indicus* on markers of cataract and the results are summarized in the Table 3.

**Table 3:** Effect of *H. indicus* on lens biochemical parameters

Parameters	Normal control	Positive control	Standard (vitamin E acetate, 100 µg/mL)	Low concentration (500 µg/mL)	High concentration (1000 µg/mL)
MDA (nmoles/100 mg)	0.9130 ± 0.073	3.893 ± 0.901^a^	1.879 ± 0.143***	3.111 ± 0.051***	2.227 ± 0.065***
AR activity (nmoles/100 mg)	29.29 ± 3.375	70.74 ± 4.39^a^	35.11 ± 0.705***	43.68 ± 4.53***	33.23 ± 1.27***
LDH (IU/L)	14.35 ± 0.685	23.29 ± 0.675^a^	14.91 ± 0.869***	13.39 ± 0.432***	12.27 ± 0.802***
SDH (μ/mg of solid)	0.2458 ± 0.151	0.851 ± 0.004^a^	0.3831 ± 0.003***	0.4567 ± 0.004***	0.3725 ± 0.003***
SOD activity (μ/mg of tissue)	2.058 ± 0.141	0.1742 ± 0.039^a^	1.442 ± 0.066***	0.9182 ± 0.069***	1.043 ± 0.053***
Catalase (U/mg of tissue)	8.286 ± 0.308	0.3876 ± 0.110^a^	7.176 ± 0.229***	3.000 ± 0.373***	6.658 ± 0.487***
GSH (nmoles/100 mg)	22.73 ± 3.010	10.60 ± 0.4727^a^	17.80 ± 0.9090*	13.67 ± 1.107^ ns^	18.67 ± 0.9236**
Calcium (mg/dl)	8.267 ± 0.4522	15.02 ± 0.4686 ^a^	13.20 ± 0.823***	14.538 ± 0.4928 ***	11.89 ± 0.5283***
Protein (mg/dl)	11.14 ± 0.3706	3.209 ± 0.4278^a^	9.232 ± 0.3560***	5.688 ± 0.4293***	8.203 ± 0.1232***
Sodium (%)	0.0222 ± 0.00015	0.0326 ± 0.00067^a^	0.02338 ± 0.0003280***	0.02893 ± 0.000421***	0.02595 ± 0.00022***
Potassium (%)	0.004167 ± 0.0003765	0.01212 ± 0.000544^a^	0.008933 ± 0.000204***	0.01005 ± 0.00064***	0.002032 ± 0.00043***
Sorbitol (µg)	3.433 ± 0.1706	7.700 ± 0.2708^a^	4.517 ± 0.2023***	6.317 ± 0.2286**	5.733 ± 0.2565***

Values are expressed as Mean ± SEM; n = 6. One-way ANOVA: p value found to be < 0.0001, considered extremely significant. Tukey–Kramer multiple comparisons test: ^a^p < 0.001 in comparison with normal control; ^b^p < 0.01 in comparison with normal control; ^c^p < 0.01 in comparison with normal control; ***p < 0.001 in comparison with positive control; **p < 0.01 in comparison with positive control; *p < 0.05 in comparison with positive control; ^ns^p > 0.05 in comparison with positive control

Malondialdehyde (MDA) levels—MDA levels were found to be high (p < 0.001) in positive control compared to normal control indicating lipid peroxidation in high galactose treated lenses. Lenses treated with *H. indicus* extract had significantly (p < 0.001) reduced MDA content at both concentrations compared with the positive control group.

Glutathione (GSH), Superoxide Dismutase (SOD), and Catalase levels—Positive control showed significantly less GSH levels, catalase, and SOD activity as compared to the normal control group. Treatment of lenses with 500 µg/mL and 1000 µg/mL of the *H. indicus* extract showed a significant elevation (p < 0.001) in catalase and SOD activity as compared to the positive control. Treatment with 1000 µg/mL of the *H. indicus* extract increased the GSH levels of lenses significantly (p < 0.01) while 500 µg/mL of extract did not show any significant change in these levels.

#### Sorbitol

The levels of sorbitol were increased significantly in galactose treated lenses in comparison with normal control. On treatment with 500 µg/mL and 1000 µg/mL of the *H. indicus* extract, these levels were decreased significantly (p < 0.01 and p < 0.001 respectively).

Levels of lactate dehydrogenase (LDH) and Sorbitol dehydrogenase (SDH)—a significant increase (p < 0.001) in the levels of LDH and SDH were observed in the lenses of the positive control group while the lenses treated with both the concentration of *H. indicus* extract showed a significant (p < 0.001) decrease in the levels of LDH and SDH.

#### Electrolytes (Ca^2+^, Na^+^, K^+^)

The positive control group showed an increase in levels of electrolytes when compared to the normal control. These levels were found to be decreased significantly (p < 0.001) in all the treatment groups.

#### Protein

Levels of protein in positive control were significantly decreased (3.209 ± 0.4278, p < 0.001) in comparison with normal control (11.14 ± 0.3706). Treatment with extract significantly increased (p < 0.001) the protein levels of the lenses.

## Discussion

A diabetic cataract is one of the most common and early developing complications in diabetic patients. Among many mechanisms that are involved in the pathogenesis of diabetic cataract, the role of the polyol pathway induced structural and biochemical changes in the diabetic lens are widely accepted. AR is the key enzyme that catalyzes the very first and most important rate-determining reaction of the polyol pathway. It is suggested that hyperglycemia activates AR and eventually polyol pathway that leads to increased build-up of sorbitol in the cell causing oxidative stress and loss of cellular proteins leading to cataract [14]. The present study demonstrates the AR inhibitory activity of *H. indicus* var. *pubescens* and its anticataract potential.

Molecular docking studies using the auto dock tool were performed to explore the binding ability of selected constituents of *H. indicus* var. *pubescens* root extract with AR enzyme. In the present molecular docking model, results demonstrated that the major constituents of *H. indicus* var. *pubescens* have interaction with AR, and the same was also confirmed by ex vivo AR inhibitory activity. This proves that the test extract and its constituents possess the potential for the control of diabetic complications like cataracts.

Many experimental models are used to study the anti-cataract potential, among which galactose induced cataract is commonly used. In the present study, galactose at a concentration of 55 mM was used to induce cataract. Galactose produces a large amount of its reduced form galactitol, inside the lens that leads to osmotic stress. Accumulation of a high concentration of polyols in the lens leads to an increase in the intracellular ionic strength resulting in excessive hydration, eventually loss of membrane integrity, and leakage of free amino acids, glutathione, and myoinositol. AR catalyzes the reduction of glucose, galactose, and xylose into the corresponding sugar alcohol, sorbitol [15]. In the present study, chemical analysis of galactose treated lenses exhibited higher AR activity which was decreased by the treatment with ethanol extract of *H. indicus* var. *pubescens* indicating its potential to inhibit AR enzyme activity.

Osmotic stress due to extensive swelling of lens fiber and accumulation of sorbitol induced stress endoplasmic reticulum resulting in the formation of free radicals. The toxic effects of the reactive oxygen species produced in the lens are neutralized by various antioxidant enzymes like SOD and catalase. However, in cataract, both of the enzyme activities are decreased making the lens more susceptible to ill effects of free radicals [16]. These changes correlate with our study findings. There was a substantial increase in levels of sorbitol and an associated decrease in antioxidant enzyme levels in the positive control. Treatment with ethanol extract of *H. indicus* var. *pubescens* decreased the levels of sorbitol and increased SOD and Catalase levels confirming the antioxidant capability of the extract. Additionally, a decreased level of sorbitol confirms that test extract inhibited AR and prevented the formation of polyols.

GSH serves as the major antioxidant in the lens and also keeps proteins in reduced form. The amount of GSH in the lens decreases almost in any type of cataract, including diabetic cataract [17]. Similarly, Lipid peroxidation, an autocatalytic process is a common cause of cell death. The by-products of lipid peroxidation are toxic compounds MDA and lipid hydroperoxides (LH) whose involvement in the pathogenesis of cataract has been suggested, mainly due to its crosslinking ability. All these above-mentioned changes were also seen in the present study and were normalized by *H. indicus var. pubescens* extract. Additionally, there was a significant inhibition of SDH and LDH activity that confirms its efficacy to retard cataract development and offer protection against the opacity of lenses.

The present study is also in agreement with the finding that incubation of lenses in the media containing high galactose (55 mM) concentration causes accumulation of Na^+^ and K^+^ resulting in hydration and swelling of the lens fibers leading to cataract formation [18]. This alteration in Na^+^ and K^+^ levels alter the protein content of the lens, leading to a decrease in water-soluble proteins which causes lens opacification. In this study, the total proteins were higher whereas, Na^+^ and K^+^ ions were lower in the extract (*H. indicus* var*. pubescens*) treated lenses. This prevention of protein loss from the lens can be accounted for the decreased lens opalescence.

The free radicals generated in the eye lens due to excess galactose concentration causes inactivation of lens Ca^2+^ ATPase leading to Ca^2+^ accumulation [19]. This enhanced calcium can cause activation of calpain-mediated proteolysis in the lens, resulting in lens opacification. The chemical analysis of the lenses of the positive control group showed a higher concentration of calcium while the *H. indicus* var*. pubescens* extract-treated lenses showed a decreased calcium level indicating the potential of *H. indicus* var. *pubescen* to protect the lenses from cataractogenesis.

The preliminary phytochemical investigation of *H. indicus* var*. pubescens* ethanol root extract revealed the presence of flavonoids and saponins. Several authors have reported antioxidant potential [9] as well as AR inhibitory activity [20] of flavonoids. Increased expression of AR and associated oxidative stress is implicated in the development of cataract. The extract contains constituents that showed significant AR inhibition ex vivo that can be accounted for their ability of AR binding as seen in docking studies. Hence, the anti-cataract potential of *H. indicus* var*. pubescens* root extract against galactose induced cataract could be due to its AR inhibitory of β-sitosterol, Hemidesmin-2, Hemidesminine, Hemidesmin-1 and antioxidant potential of flavonoids present in the extract.

## Conclusion

The present study suggests that the ethanol root extract of *H. indicus* var*. pubescens* was found to have significant anti-cataract activity against galactose induced cataract in isolated goat lens. This anti-cataract potential is mainly accounted for the antioxidant activity and AR inhibitory activity of β-sitosterol, Hemidesmin-2, Hemidesminine, and Hemidesmin-1 as confirmed by molecular docking studies. However, in vivo studies could also be performed with the extract as well as isolated components to substantiate the claim.

## Materials and Methods

### Collection of Plant Material

The roots of *H. indicus* var. *pubescens* were collected from Thiruvananthapuram district, Kerala, India. The plant material was identified and authenticated by a taxonomist and the herbarium specimen of the same was prepared and deposited in the herbarium of the Department of Pharmacognosy, Faculty of Pharmacy, MSRUAS, Bengaluru.

### Preparation of Plant Extract

*H. indicus* var. *pubescens* roots were dried at room temperature, coarsely powdered was extracted in a soxhlet apparatus with solvent 95% ethanol at 40 °C. The extract was filtered using Whatman filter paper and the solvent was evaporated to obtain the dried and concentrated extract that was stored in an air-tight container for further use [11].

### Phytochemical Studies

The prepared root extract was subjected to preliminary phytochemical screening for the presence of various phytoconstituents [21].

### Molecular Docking Studies

#### Ligand and Protein Preparation

β-sitosterol, hemidesminine, hemidesmin-1 and 2 (major constituent of *H. indicus*) were selected for docking analysis. The chemical structure of β-sitosterol was obtained using the Pub chem database (http://www.ncbi.nlm.nih.gov/pccompound). Marvin Sketch 17.4.3 was used for generating the structure of hemidesminine, hemidesmin-1 and 2. After assessing several co-crystallised structures of AR protein at RCBS protein data bank (http://www.rcsb.org/pdb), the best ligand-bound complexes with best binding interactions with ligands was used for the study (PDB ID: 1US0).

#### Docking Protocol

Auto Dock Tools (ADT) version 1.5.6 and Autodock version 4.2 programs from Scripps Research Institute were used to perform docking analysis [22]. Preparation of protein and ligands and grid box creation were completed using the Graphical User Interface program of ADT. Polar hydrogen charges and Kollman charges were assigned and atomic solvation parameters were added. AutoDock saved the prepared file in PDBQT format. AutoGrid was used for the preparation of the grid map using a grid box. The grid size was set to 126 × 126 × 126 xyz points with a grid spacing of 0.375 Å and the grid center was designated at dimensions (x, y, and z): 13.189, 1.064, and 2.796. Lamarckian Genetic Algorithm was used for docking conformational search with a population of 100 individuals with a mutation rate of 0.02 were evolved for 1000 generations. A cluster analysis based on root mean square deviation (RMSD) values, with reference to the starting geometry, was performed and the conformation with the lowest energy of the more populated cluster was considered. The pose with the lowest energy of binding or binding affinity was extracted and aligned with receptor structure. PyMol molecular viewer (The PyMOL Molecular Graphics System, Version 1.5.0.4 Schrödinger, LLC) and Discovery Studio Visualizer (Discovery Studio Visualizer ver. 20.1.0.19295) [23] was used for analyzing the ligand–protein interactions of the compounds.

### Ex Vivo Anti-Cataract Activity

#### Preparation of Lens Culture

Fresh goat lenses were obtained from the nearby slaughterhouse and stored at 0–4 °C. Lenses were removed by extra-capsular extraction and incubated in artificial aqueous humor (NaCl—140 mM, KCl—5 mM, MgCl_2_—2 mM, NaHCO_3_—0.5 mM, NaH_2_PO_4_—0.5 mM, CaCl_2_—0.4 mM and Glucose 5.5 mM) at room temperature and pH 7.8 for 72 h. To prevent bacterial contamination, Penicillin 32 mg%, and streptomycin 250 mg% were added to the culture media. Galactose in a concentration of 55 mM was used to induce cataract [24].

#### Experimental design

A total of 30 lenses were divided into five groups, with a total of 6 lenses in each group. The lenses were grouped as;

Group I—Normal control (lens subjected to only artificial aqueous humor).

Group II—Positive or toxic control (Lens subjected to artificial aqueous humor + 55 mM Galactose).

Group III—Standard drug-treated (Lens subjected to artificial aqueous humor + 55 mM Galactose + Vitamin E acetate − 100 µg/mL).

Group IV—Extract at low concentration (Lens subjected to artificial aqueous humor + 55 mM Galactose + Low concentration, 500 µg/mL).

Group V—Extract at high concentration (Lens subjected to artificial aqueous humor + 55 mM Galactose + High concentration, 1000 µg/mL).

#### Lens Morphology

To study the morphology of lens, the lenses were placed on a graph sheet and the number of squares visible through the lenses was observed as a measure of lens opacity. Changes like haziness, swelling, or other morphological disruption were also noted. The grading for the cataract changes were done following the criteria [25] given in Table 4.

**Table 4:** Grades for cataract changes

Grade	Description	Details
0	No changes	Visible grid lines, lens outline and shape preserved
1	Mild	Visible grid lines, minimal lens swelling, lens outline and shape preserved
2	Moderate	Faintly visible grid lines, lens swelling present
3	Moderate to severe	Almost obstructed grid lines, lens outline and shape damaged
4	Severe	Invisible grid lines, distorted lens shape and outline, mature cataract about to rupture

#### Preparation of Lens Homogenate

After 72 h of incubation, lenses from each group were separated and 10% w/v homogenate was prepared in 50 mM phosphate buffer (pH 7.4). The homogenate was centrifuged at 10,000×*g* at − 4 °C for 20 min using a cooling centrifuge. The supernatant was collected and biochemical parameters like AR activity [26], lipid peroxidation measured as MDA [27], GSH, SOD, and catalase [28], calcium [29, 30], sorbitol and SDH [31, 32], LDH [33], total protein (GenX Total protein kit) were estimated.

#### Statistical Analysis

The results of parametric data are expressed as mean ± S.E.M and were tested with one-way ANOVA followed by the Tukey–Kramer multiple comparison test.
